# Use of Antihypertensive Drugs and Ischemic Stroke Severity – Is There a Role for Angiotensin-II?

**DOI:** 10.1371/journal.pone.0166524

**Published:** 2016-11-15

**Authors:** Wen Yea Hwong, Michiel L. Bots, Sharmini Selvarajah, Zariah Abdul Aziz, Norsima Nazifah Sidek, Wilko Spiering, L. Jaap Kappelle, Ilonca Vaartjes

**Affiliations:** 1 National Clinical Research Centre, Kuala Lumpur General Hospital, Kuala Lumpur, Malaysia; 2 Julius Center for Health Sciences and Primary Care, University Medical Center Utrecht, Utrecht, the Netherlands; 3 Department of Neurology, Hospital Sultanah Nur Zahirah, Terengganu, Malaysia; 4 Department of Pharmacy, Hospital Sultanah Nur Zahirah, Terengganu, Malaysia; 5 Department of Vascular Medicine, University Medical Center Utrecht, Utrecht, the Netherlands; 6 Department of Neurology and Neurosurgery, Brain Center Rudolf Magnus, University Medical Center Utrecht, Utrecht, the Netherlands; Ehime University Graduate School of Medicine, JAPAN

## Abstract

**Background:**

The increase in angiotensin II (Ang II) formation by selected antihypertensive drugs is said to exhibit neuroprotective properties, but this translation into improvement in clinical outcomes has been inconclusive. We undertook a study to investigate the relationship between types of antihypertensive drugs used prior to a stroke event and ischemic stroke severity. We hypothesized that use of antihypertensive drugs that increase Ang II formation (Ang II increasers) would reduce ischemic stroke severity when compared to antihypertensive drugs that suppress Ang II formation (Ang II suppressors).

**Methods:**

From the Malaysian National Neurology Registry, we included hypertensive patients with first ischemic stroke who presented within 48 hours from ictus. Antihypertensive drugs were divided into Ang II increasers (angiotensin-I receptor blockers (ARBs), calcium channel blockers (CCBs) and diuretics) and Ang II suppressors (angiotensin-converting-enzyme inhibitors (ACEIs) and beta blockers). We evaluated stroke severity during admission with the National Institute of Health Stroke Scale (NIHSS). We performed a multivariable logistic regression with the score being dichotomized at 15. Scores of less than 15 were categorized as less severe stroke.

**Results:**

A total of 710 patients were included. ACEIs was the most commonly prescribed antihypertensive drug in patients using Ang II suppressors (74%) and CCBs, in patients prescribed with Ang II increasers at 77%. There was no significant difference in the severity of ischemic stroke between patients who were using Ang II increasers in comparison to patients with Ang II suppressors (OR: 1.32, 95%CI: 0.83–2.10, p = 0.24).

**Conclusion:**

In our study, we found that use of antihypertensive drugs that increase Ang II formation was not associated with less severe ischemic stroke as compared to use of antihypertensive drugs that suppress Ang II formation.

## Introduction

Although there are no differences in the extent of blood pressure lowering between types of antihypertensive drugs, certain classes of drugs are postulated to potentially confer a greater amount of cerebroprotection than others.[1] Fournier et al in 2004 [1] has reinforced the possible role of angiotensin II (Ang II) in stroke protection after it was first proposed by Brown and Brown [2] in 1986. Supported by experimental evidence [3,4], several neuroprotective pathways that arise from the increase in Ang II formation are identified; the most acknowledged mechanism is via increase in angiotensin-II-type-2 (AT2) receptor stimulation.[5] The stimulation of AT2 receptors is believed to increase neuronal resistance to ischemia and promote collateral circulation recruitments after an ischemic event.[1,6] In addition, increase in Ang II formation leads to similar neuroprotective functions via upregulation of Angiotensin-(1–7) and stimulation of Mas receptors.[5]

Angiotensin-1 receptor blockers (ARBs), calcium channel blockers (CCBs) and diuretics are antihypertensive drugs that increase Ang II formation and thus, these drugs are believed to have neuroprotective properties against ischemic stroke. This hypothesis was initially proposed by Fournier et al [1] after consistent findings of a reduction in stroke risk with ARBs, CCBs and diuretics in numerous clinical trials and similarity in terms of mechanism of Ang II formation in these drugs. With an unopposed formation of Ang II and a selective inhibition of angiotensin-II-type-I (AT1) receptors, the administration of ARBs increases AT2 receptor stimulation. The mechanism is slightly different for diuretics and CCBs where both drugs act by promoting renin secretion which subsequently increase Ang II formation. Conversely, both angiotensin-converting-enzymes inhibitors (ACEIs) and beta blockers suppress renin secretion and thus, decrease the formation of Ang II.[6]

Two clinical trials that looked into the effects of antihypertensive drugs on functional impairment post-stroke reported contrasting findings to the positive results obtained in experimental studies.[7,8] The Prevention Regimen for Effectively Avoiding Second Stroke Trial (PROFESS), which compared an ARBs (telmisartan) with a placebo showed that functional impairment 3 months post-stroke between the groups was not different.[7] In the Scandinavian Candersartan Acute Stroke Trial (SCAST), functional impairment 6 months post-stroke was worse in the ARBs (candersartan)-treated group compared to placebo.[8] However, possible neuroprotective benefits of the drugs via increase in Ang II formation could not be identified from both trials because both treatment arms were treated in addition to standard antihypertensive drugs. Combinations of drugs that increase and suppress Ang II formation could possibly be prescribed to both groups and this hampers comparisons. In addition, the limited number of observational studies that investigated possible translation of these effects into improvement in patient outcomes showed inconsistent results.[9–14]

Therefore, the current study was conducted to assess the relationship between types of antihypertensive drugs used prior to an ischemic stroke event and the severity of ischemic stroke. We hypothesized that use of antihypertensive drugs that increase Ang II formation prior to the stroke onset would be associated with less severe ischemic stroke than use of antihypertensive drugs that suppress Ang II formation.

## Methods

### Cohort enrollment and exposure measures

Data was retrieved from the Malaysian National Neurology Registry.[15] The registry identifies stroke patients from 14 participating public hospitals in Malaysia. Between July 2009 and December 2014, a total of 7592 patients were registered. Collection of data for this registry followed local routine clinical practice. Depending on availability, information on co-morbidities and drug prescriptions were verified with patients’ past medical records from respective general practitioners.

For the present cross-sectional study, we included patients who were: 1) admitted with first event of ischemic stroke or transient ischemic attack (TIA); 2) arrived at the hospitals within 48 hours from ictus; and 3) diagnosed with hypertension and were receiving antihypertensive drugs before the onset of their stroke events. The diagnosis of ischemic stroke is based on patients’ clinical signs and symptoms and further confirmed via interpretation of computed tomographic (CT) imaging.

As for use of antihypertensive drugs prior to the stroke event, the drugs were divided into 2 categories, namely antihypertensive drugs that increase the formation of Ang II (ARBs, CCBs and diuretics) and antihypertensive drugs that suppress Ang II formation (ACEIs and beta blockers). In subsequent sections of the paper, antihypertensive drugs that increase Ang II formation will be described as Ang II increasers and likewise, the term Ang II suppressors will be used to describe antihypertensive drugs that suppress Ang II formation.[9] We excluded patients who were treated with a combination of both groups and patients who were on antihypertensive drugs other than ACEIs, ARBs, CCBs, beta blockers and diuretics.

Several potential confounding factors were identified. We included demographic characteristics: age, sex, educational level and ethnic group; co-morbidities: diabetes mellitus, dyslipidemia, atrial fibrillation and heart diseases; concomitant drugs: lipid-lowering drugs, antiplatelet and anticoagulants; and lifestyle related confounders: smoking status and obesity. More information on the operationalization of these confounders can be found in S1 Table.

### Outcome measure

Ischemic stroke severity was measured by the National Institute of Health Stroke Scale (NIHSS) during admission. Patients were scored with integers ranging from 0 to 42. The total score is a sum of scores from 15 domains of neurological measurements.[16] As there is no standardized cut-offs for this score, we opted to dichotomize the score at 15, which is a commonly used cut-off value. A score of less than 15 was categorized as less severe stroke. In view of the overall median NIHSS score of 5(IQR: 2; 12) in our sample, we repeated the analysis with another cut-off value of 7. Both cut-off values were verified by Adams et al. who reported an excellent recovery at 3 months in 90% of patients with NIHSS score lower than 7 and a less than 20% chance of recovery for patients with NIHSS score greater than 15.[17]

### Ethical consideration

Ethical approval was obtained from the Malaysian Ministry of Health’s research ethics committee (ID: NMRR 08-1631-3189). The approval includes data collection and use of data for secondary analysis. With a waiver of informed consent, a public notice is displayed at all participating sites and participants have the option to opt out.

### Statistical analysis

The proportion of missing data was observed to range from 0.07% (variable: ethnic group) to 45% (variable: smoking status). As the pattern of missing data that occurred in the variables used for inclusion criteria were missing at random, we performed multiple imputation with m = 10 (number of imputations) prior to the inclusion of patients for actual analysis. Post imputation, patients who did not fulfill the inclusion criteria were removed from the analysis. Here, we reported results from the imputed model because it yields least bias results[18] while maintaining the power of the study. For counts and descriptive statistics, we produced a ‘best fitted dataset’ by taking modes for each imputed variable per individual patient. In the event of multiple modes, the mode with highest value was taken. Multiple imputation was conducted with R version 3.1.1.[19]

Next, multivariable logistic regressions for scores at both cut-off values were performed to determine the relationship between types of antihypertensive drugs and ischemic stroke severity. Odds ratio with its corresponding 95% confidence intervals were reported. We also performed additional analyses where first, the NIHSS score was kept in its original form as count data. We conducted a Poisson regression which was subsequently converted to a negative binomial regression to account for overdispersion. Second, we performed another multivariable logistic regression looking into individual effects of antihypertensive drugs on ischemic stroke severity. For the latter analysis, only patients on monotherapy prescription were selected to allow for quantification of effect size for each drug.

All model assumptions were checked and no obvious violations were observed. Statistical analysis was performed using Stata version 13.0.[20] Significance level was taken at p<0.05.

## Results

### Patient characteristics

**Fig 1:**
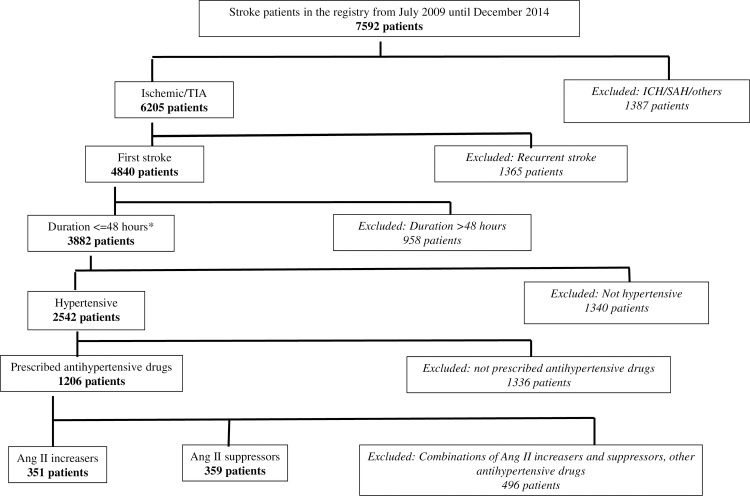
Flowchart for inclusion of patients (n = 710). *duration from onset of stroke symptoms to hospital arrival, TIA: transient ischemic attack, ICH: intracranial hemorrhage, SAH: subarachnoid hemorrhage, Ang II: Angiotensin II.

Selection of patients for inclusion in this study is displayed in Fig 1. Of 710 patients who were included in the analysis, the proportion of patients who were treated with Ang II increasers and Ang II suppressors were almost equal; 49.4% (n = 351) for the former and 50.6% (n = 359) in the latter (Table 1). Demographic characteristics were similar across both groups. Only 2% of patients in the Ang II increasers group had a diagnosis of TIA. The proportion was doubled in patients who were receiving Ang II suppressors. Seventy-four percent of patients with Ang II suppressors took ACEIs whereas in the group of Ang II increasers, CCBs were the most commonly prescribed antihypertensive drugs (77%). In both groups, 80% of patients had monotherapy prescription of antihypertensive drugs.

**Table 1:** Baseline Characteristics by Types of Antihypertensive Drugs.*

Characteristics	Ang II increasers (ARBs, CCBs, Diuretics)	Ang II suppressors (ACEIs, Beta blockers)	p-value
	n = 351	n = 359	
Mean age(years) ± SD	64 ± 12	63 + 12	0.26
Sex, n (%)			
Men	168 (48)	167 (47)	
Women	183 (52)	192 (53)	0.72
Ethnic group, n (%)			
Malay	288 (82)	294 (82)	
Non-Malay	63 (18)	65 (18)	0.96
Educational level, n (%)			0.08
Nil	74 (21)	51 (14)	
Primary	150 (43)	179 (50)	
Secondary	110 (31)	114 (32)	
Tertiary	17 (5)	15 (4)	
Proportion of antihypertensive drugs, n (%)			-
ACEIs	-	266 (74)	
Beta blockers	-	165 (46)	
ARBs	34 (10)	-	
CCBs	271 (77)	-	
Diuretics	123 (35)	-	
Co-morbidities, n (%)			
Diabetes Mellitus	186 (53)	211 (59)	0.12
Dyslipidemia	132 (38)	114 (32)	0.10
Atrial Fibrillation	16 (5)	14 (4)	0.66
Heart diseases	41 (12)	80 (22)	< 0.001
Hyperuricemia	22 (6)	6 (2)	0.002
Life-style factors, n (%)			
Obesity	26 (7)	41 (11)	0.07
Smoking status			0.95
Never smoked	214 (61)	221 (62)	
Previous smoker (quit >30 days)	61 (17)	59 (16)	
Current smoker	76 (22)	79 (22)	
Excessive alcohol intake	5 (1)	4 (1)	0.71
Concomitant medication, n (%)			
Lipid-lowering drugs	135 (38)	197 (55)	< 0.001
Antiplatelet	94 (27)	143 (40)	< 0.001
Anticoagulants	8 (2)	7 (2)	0.76
Mean Systolic BP upon arrival (mmHg) ± SD	163 + 30	170 + 31	0.002
Mean Diastolic BP upon arrival (mmHg) ± SD	89 + 17	93 + 19	0.002
Median Time of onset to arrival in hospital(hours) (IQR)	7 (3; 17)	6 (2; 18)	0.37
Median NIHSS Score (IQR)	5 (2;11)	5 (2;13)	0.77
TOAST classification for ischemic stroke subtypes, n (%)			0.03
Large vessel	178 (51)	154 (43)	
Lacunar	123 (35)	137 (38)	
Cardioembolic	15 (4)	9 (3)	
Others (Determined, Undetermined)	35 (10)	59 (16)	

*Ang II: Angiotensin-II-; ACEIs: angiotensin-converting-enzymes inhibitors; ARBs: angiotensin-1 receptor blockers; CCBs: calcium channel blockers; SD: standard deviation; IQR: interquartile range; BP: blood pressure; TOAST: Trial of Org 10172 in Acute Stroke Treatment

The proportion of patients with history of heart diseases in the group with Ang II suppressors was significantly higher than those who received Ang II increasers; 22% for the former and 12% for the latter (p<0.001). Similarly, higher proportions of obese and diabetic patients were observed in patients who received Ang II suppressors; despite the increase being not statistically significant. In contrast, only 2% of patients who were using Ang II suppressors had hyperuricemia whereas in patients who took Ang II increasers, the proportion was 6% (p<0.002).

In terms of concomitant drugs, significantly higher proportions of other cardioprotective drugs were being prescribed to patients with Ang II suppressors; 55% versus 38% received a lipid lowering drug (p<0.001) and 40% versus 27% were prescribed with an antiplatelet drug (p<0.001).

Furthermore, patients who received Ang II suppressors were reported to have significantly higher blood pressure levels (p<0.002) with a mean systolic measurement of 170mmHg (SD: 31) and a mean diastolic of 93mmHg (SD: 19). Although the median NIHSS score between both groups did not differ, there were variations in the unadjusted median NIHSS score for each individual antihypertensive drug; lowest median score observed was for patients with ARBs at 2 (IQR: 1; 10), followed by ACEIs and CCBs (median: 5; IQR: 2; 11), beta blockers (median: 6; IQR: 2; 11) and diuretics (median: 8; IQR: 3; 16). Overall, subtypes of ischemic stroke according to TOAST classification was found to be significantly different between the two exposure groups (p<0.03). There were higher percentages of patients with ischemic stroke of large vessel atherosclerosis (51%) and cardioembolic (4%) origin in Ang II increasers group in comparison to Ang II suppressors group (43% for large vessel atherosclerosis and 3% for cardioembolic, respectively).

### Association between types of antihypertensive drugs and ischemic stroke severity

As shown in Table 2, there was no significant difference in the severity of ischemic stroke between patients who were treated with Ang II increasers in comparison to Ang II suppressors (OR: 1.32; 95%CI: 0.83–2.10). Dichotomizing NIHSS score at 7 also gave a statistically insignificant odds ratio of 0.99 (95%CI: 0.70–1.39). By having the score in its original count measure (Table 3), the effects of Ang II increasers compared to Ang II suppressors on ischemic stroke severity were consistently insignificant with a rate ratio of 1.05 (95%CI: 0.89–1.24).

**Table 2:** Association between types of antihypertensive drugs and ischemic stroke severity (Dichotomized score).*^†^

Dichotomization of NIHSS score	Types of antihypertensive drugs	Odds Ratio (OR) with 95% CI	p-value
Dichotomized at 7	Ang II suppressors	*1*.*00 (reference)*	-
	Ang II increasers	0.99 (0.70–1.39)	0.95
Dichotomized at 15	Ang II suppressors	*1*.*00 (reference)*	-
	Ang II increasers	1.32 (0.83–2.10)	0.24

*Adjusted for potential confounders including demographic characteristics: age, sex, educational level, ethnic group; co-morbidities: diabetes mellitus, dyslipidemia, atrial fibrillation, heart diseases, hyperuricemia; lifestyle related factors: obesity, smoking status; and concomitant drugs: anticoagulants, antiplatelet, lipid-lowering drugs.

^†^Ang II suppressors: antihypertensive drugs that suppress angiotensin II formation (ACEIs, Beta blockers); Ang II increasers: antihypertensive drugs that increase angiotensin II formation (ARBs, CCBs, Diuretics); NIHSS: National Institute of Health Stroke Scale.

**Table 3:** Association between types of antihypertensive drugs and ischemic stroke severity (Score in count measure).*^†^

Types of antihypertensive drugs	Rate ratio with 95% CI	p-value
Ang II suppressors	*1*.*00 (reference)*	-
Ang II increasers	1.05 (0.89–1.24)	0.59

*Adjusted for potential confounders including demographic characteristics: age, sex, educational level, ethnic group; co-morbidities: diabetes mellitus, dyslipidemia, atrial fibrillation, heart diseases, hyperuricemia; lifestyle related factors: obesity, smoking status; and concomitant drugs: anticoagulants, antiplatelet, lipid-lowering drugs.

^†^Ang II suppressors: antihypertensive drugs that suppress angiotensin II formation (ACEIs, Beta blockers); Ang II increasers: antihypertensive drugs that increase angiotensin II formation (ARBs, CCBs, Diuretics).

Further analysis looking into individual effects of antihypertensive drugs in comparison to ACEIs (reference) found similar results (S2 Table). All observed effects were statistically insignificant.

## Discussion

We found no associations between types of antihypertensive drugs and the severity of ischemic stroke. Findings from our study showed that use of Ang II increasers prior to a stroke event was not associated with a reduction in ischemic stroke severity when compared to use of Ang II suppressors.

Our findings are in line with results from a previous study by Ovbiagele et al in 2005.[9] They reported no significant difference (p value = 0.12) in the severity of ischemic stroke between patients who were treated with Ang II increasers (ARBs, CCBs, and thiazide diuretics) and Ang II suppressors (ACEIs and beta blockers). With a larger sample size (n = 710 versus n = 65), we were able to appropriately adjust for more confounding factors to reduce potential bias in the investigated association. Furthermore, we included all types of diuretics in our analysis instead of only thiazide diuretics. Our rationale is that the hypothesis underlying the mechanism that increases angiotensin II formation via stimulation of renin secretion did not differentiate between thiazide diuretics and non-thiazide diuretics.[1,6] Besides that, we conducted an additional analysis looking into the effect size of each antihypertensive drug. The extent of cerebroprotection via increase or suppression of Ang II formation may differ between drugs and therefore we postulated that quantification of their individual effects on ischemic stroke severity might vary. Similar to our main results, we did not find any association between each antihypertensive drug and ischemic stroke severity. Having said that, the statistical significance for ARBs would have to be interpreted with caution because there were only a few patients who received ARBs as monotherapy.

Variations in findings from other previous studies could be attributed to many factors. First, observational studies with causal aims are prone to bias from confounding by indication. One way to reduce this type of bias is to select patients with similar indications of treatment for comparison.[21] Thus, we restricted our sample to patients who were diagnosed with hypertension and who took antihypertensive drugs prior to their stroke events. With this selection, comparisons between exposure groups are relatively more equal. Discrepancies in the results from previous studies could arise because they[11–14] included hypertensive patients without antihypertensive drugs and patients who were not diagnosed hypertensive, with or without antihypertensive drugs. Inclusion of these patients tend to affect the validity of the results because antihypertensive drugs could be prescribed for other indications that were unknown in the studies. Moreover, normotensive and hypertensive patients without prescription of antihypertensive drugs could have different controls of blood pressure when compared to hypertensive patients who were treated with antihypertensive drugs. Unless the severity of their hypertension was accounted for, allowing for comparisons between these groups of patients could lead to bias.

There were also differences across studies in terms of outcome measure. Miyamoto et al,[10] found that patients who were treated with pre-stroke ARBs were 3.81 times (95%CI: 1.11–13.07) more likely to have a less severe stroke when compared to those who did not receive pre-stroke ARBs. The outcome however, was assessed during hospital discharge. We took stroke severity during admission due to the possibility of changes in the association between exposure and outcome in patients who received thrombolytic therapy and those who had an adjustment of prescription during hospitalization. In addition, stroke severity was also measured with different types of scores, which complicate comparisons between studies. Apart from the NIHSS score, scores such as the Canadian Neurological Scale[11], the Modified Rankin Score and the Barthel Index[10] were also used. Although there has been arguments on the accuracy of the NIHSS score in patients with posterior circulation infarcts[22], this score was found to be superior in comparison to other stroke severity scores for prognostication.[23] Studies that have assessed ischemic stroke severity with the NIHSS score also had differences with regards to cut-off values and their choice of analysis depending on their interpretation of the nature of the outcome measure. To accommodate for this, we performed our analyses with two verified cut-off values. The cut-off value of 7 is often used to discriminate between mild and moderate stroke while at 15, patients with moderate and severe stroke can be distinguished. Regardless of the cut off values, odds ratios were consistently near to the null value with confidence intervals that crosses 1. Similarly, we tried to compare our analysis with studies that have assessed the NIHSS score as a continuous measure.[9,14] Instead of a linear regression, we performed an additional negative binomial regression (an option to Poisson regression) which we believed was more appropriate as the outcome measure is a score bounded at the value 0 with a ceiling value of 42. Results were consistent with our main analysis.

The main strength of our study lied in our methods. Besides the abovementioned adjustments, we conducted multiple imputation to minimize the amount of bias that may occur as a result of missing data. Our study sample was also moderate in size in comparison to previous studies. Some of our limitations include the lack of information on adherence to treatment as well as control of blood pressure levels. Despite performing a multivariable analysis, we were not able to adjust for possible residual confounders that were not collected as part of the registry.

## Clinical Implications

The hypothesis of a reduction in the risk of ischemic stroke and its severity via increase in Ang II formation stirred up interest in many because there has been a long standing interest in finding potential added benefits of antihypertensive drugs beyond their blood pressure lowering effects. ARBs are more than often thought to be the drug of choice for cerebroprotection due to its dual action of AT1 receptor blockade and increase in AT2 receptor stimulation.[24] The increase in Ang II formation via the promotion of renin secretion in CCBs and diuretics does not block disruptive events that occur from the simultaneous simulation of AT1 receptors. Nevertheless, the indirect AT2 receptor stimulation and the presence of other neuroprotective pathways that results from the increase in Ang II formation is believed to justify the possible neuroprotective contributions in CCBs and diuretics. By proving this relationship, this may potentially change a physician’s choice of antihypertensive drugs especially in patients with high risk of ischemic stroke. However in our study, we were not able to translate the positive response observed in experimental models into benefits in clinical use to reduce ischemic stroke severity. Findings from our study showed that collectively, use of Ang II increasers was not associated with a less severe ischemic stroke as compared to Ang II suppressors.

We acknowledge the relatively smaller proportion of patients receiving ARBs in the Ang II increasers group in comparison to CCBs and diuretics. However, the proportion of ARBs is not of importance if increase in Ang II formation as a group does reduce the severity of an ischemic stroke. Now that our results showed otherwise, we would like to postulate a few reasons that could have possibly lead to these findings. Apart from a possible true no-effect of the increase in Ang II formation on ischemic stroke severity in human patients, the validity of grouping antihypertensive drugs based on the formation of Ang II should perhaps be reconsidered. As mentioned earlier, there are several possible neuroprotective pathways with the increase in Ang II formation and thus, the extent of cerebroprotection may differ between individual drugs. Although our attempt to investigate this relation did not result in any significant associations, future research in a population with a larger number of patients treated with ARBs will be desirable to confirm what we have found.

## Conclusion

In summary, we found that use of antihypertensive drugs that increase Ang II formation was not associated with less severe ischemic stroke as compared to use of antihypertensive drugs that suppress Ang II formation.

## Supporting Information

S1 Table Operationalization of confounders. (DOCX)

S2 Table Association between types of antihypertensive drugs and ischemic stroke severity (Individual effects). (DOCX)
