# Supplementary material for: Use of Antihypertensive Drugs and Ischemic Stroke Severity – Is There a Role for Angiotensin-II?
Source: PLoS One. 2016 Nov 15;11(11):e0166524. doi: 10.1371/journal.pone.0166524 (PMC5112945; doi:10.1371/journal.pone.0166524)
Supplement: S1 Table — (DOCX) [file pone.0166524.s001.docx]

Supporting Information

S1 Table. Operationalization of confounders

| Characteristics | Type of variable | Operationalization |
| --- | --- | --- |
| Age | Numerical | measured in years from the date of birth to the date of admission |
| Sex | Nominal | dichotomous variable: men and women |
| Ethnic group | Nominal | dichotomous variable: Malay and non-Malay |
| Education level | Nominal | measured by the highest education attained: none, primary education, secondary education and tertiary education |
| Co-morbidities |  |  |
| Diabetes Mellitus | Nominal | documented history of diabetes mellitus type 2 prior to stroke event: yes and no |
| Dyslipidemia | Nominal | documented history of dyslipidemia prior to stroke event: yes and no |
| Atrial Fibrillation | Nominal | documented history of atrial fibrillation prior to or during stroke event: yes and no |
| Heart diseases | Nominal | documented history of any heart diseases including ischemic heart disease, congestive heart failure, rheumatic heart disease or heart valve diseases prior to stroke event: yes and no |
| Hyperuricemia | Nominal | documented history of hyperuricemia prior to stroke event: yes and no |
| Life-style factors |  |  |
| Obesity | Nominal | measured by body mass index of >25kg/m2: yes and no (Asian classification for body mass index from World Health Organization) |
| Smoking status | Nominal | categorized into 3 statuses of smoking: 1 for never smoked before prior to the stroke event; 2 for former smoking history (quitted more than 30 days); and 3 for currently smoking |
| Concomitant drugs |  |  |
| Anticoagulants | Nominal | concomitant medication prior to the stroke event coded with ATC code B01A except B01AC: yes and no |
| Antiplatelet | Nominal | concomitant medication prior to the stroke event coded with ATC code B01AC: yes and no |
| Lipid-lowering drugs | Nominal | concomitant medication prior to the stroke event coded with ATC code C10: yes and no |
