# Supplementary material for: Use of Antihypertensive Drugs and Ischemic Stroke Severity – Is There a Role for Angiotensin-II?
Source: PLoS One. 2016 Nov 15;11(11):e0166524. doi: 10.1371/journal.pone.0166524 (PMC5112945; doi:10.1371/journal.pone.0166524)
Supplement: S2 Table — (DOCX) [file pone.0166524.s002.docx]

Supporting Information

S2 Table. Association between types of antihypertensive drugs and ischemic stroke severity (Individual effects)*†

| Dichotomization of NIHSS score | Types of antihypertensive drugs | | Odds Ratio (OR) with 95% CI | p-value |
| --- | --- | --- | --- | --- |
| Dichotomized at 7 | Ang II suppressors | ACEIs | *1.00 (reference)* | - |
|  |  | Beta-blockers | 1.76 (0.91 - 3.39) | 0.09 |
|  | Ang II increasers | ARBs | 0.91 (0.21 - 3.90) | 0.90 |
|  |  | CCBs | 1.20 (0.75 - 1.91) | 0.46 |
|  |  | Diuretics | 1.50 (0.79 - 2.88) | 0.22 |
| Dichotomized at 15 | Ang II suppressors | ACEIs | *1.00 (reference)* | - |
|  |  | Beta-blockers | 1.18 (0.49 - 2.89) | 0.71 |
|  | Ang II increasers | ARBs | 2.10 (0.38 - 11.54) | 0.39 |
|  |  | CCBs | 1.41 (0.74 - 2.66) | 0.29 |
|  |  | Diuretics | 2.01 (0.90 - 4.51) | 0.09 |

*Only monotherapy prescription was included in this analysis (n=570 with a variation of sample size from n=9(ARBs) to n=207(CCBs)). Models were adjusted for potential confounders including demographic characteristics: age, sex, educational level, ethnic group; co-morbidities: diabetes mellitus, dyslipidemia, atrial fibrillation, heart diseases, hyperuricemia; lifestyle related factors: obesity, smoking status; and concomitant drugs: anticoagulants, antiplatelet, lipid lowering drugs.

^†^Ang II suppressors: antihypertensive drugs that suppress Angiotensin II formation (ACEIs, Beta blockers); Ang II increasers: antihypertensive drugs that increase Angiotensin II formation (ARBs, CCBs, Diuretics); NIHSS: National Institute of Health Stroke Scale.
